# Supplementary material for: FRS2-independent GRB2 interaction with FGFR2 is not required for embryonic development
Source: Biol Open. 2023 Jul 27;12(7):bio059942. doi: 10.1242/bio.059942 (PMC10399203; doi:10.1242/bio.059942)
Supplement: Supplementary information [file biolopen-12-059942-s1.pdf]

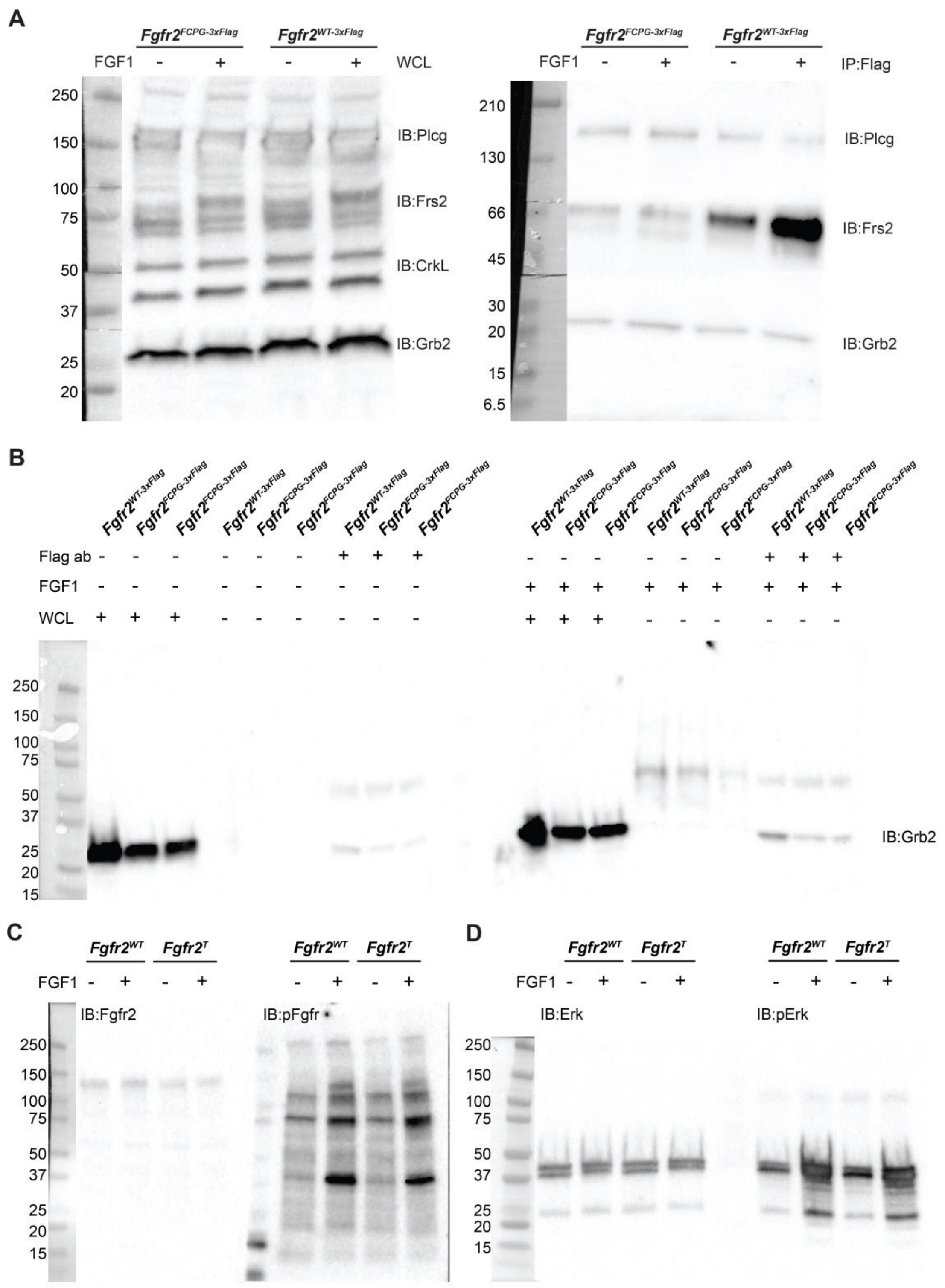


Biology Open • Supplementary information

**Fig. S1. Generation of novel *Fgfr2$^{T}$* and *Fgfr2$^{FCPGT}$* allele in vivo.** A) Uncropped Western blots from Figure 1. Molecular marker used in the left blot was Bio-Rad Precision Plus Protein Dual Color Standards. The marker used on the right blot was Bio-Rad Prestained SDS-PAGE Broad Range Standards. B) GRB2 is bound to Fgfr2WT-3xFlag and Fgfr2FCPG-3xFlag in both the absence and presence of FGF1. Pull down of GRB2 is not observed in the absence of FLAG antibody, using only IgG. Molecular marker used in this blot was Bio-Rad Precision Plus Protein Dual Color Standards. C) Homozygous Fgfr2T embryonic fibroblasts show comparable levels of FGFR2 expression and exhibit similar levels of pFGFR in response to FGF1 treatment, compared to wild type. Molecular marker used in this blot was Bio-Rad Precision Plus Protein Dual Color Standards. D) Homozygous Fgfr2T embryonic fibroblasts show similar pErk activation in response to FGF1 treatment compared to wild type. Molecular marker used in this blot was Bio-Rad Precision Plus Protein Dual Color Standards.

**Table S1. Oligonucleotides used in this study**

| | |
|---|---|
| T_sgRNA | 5′:CAGACAGGGTTCATAAGGCATGG:3′ |
| T_ssODN | 5′:TTACCCCGACACAAGGAGCTCTTGTTCTTCAGGGGACGATTCTGTGTTTTCTC CAGACCCGATGCCATACGAGCCTTGTCTGCCTCAGTGACCATGGATAAACGGCA GTGTTAAAACATGAGTGAATGTGTCTTCCTGTCCCCAAACAGGACAGCAC:3′ |
| T_for_primer | 5′:TTCAGGGTAGCCTGGAGTGA:3′ |
| T_rev_primer | 5′:TGCGGCTGTCCACTTATCAG:3′ |

Biology Open • Supplementary information

**Dataset 1. *Fgfr2*$^{T}$ sequence.**

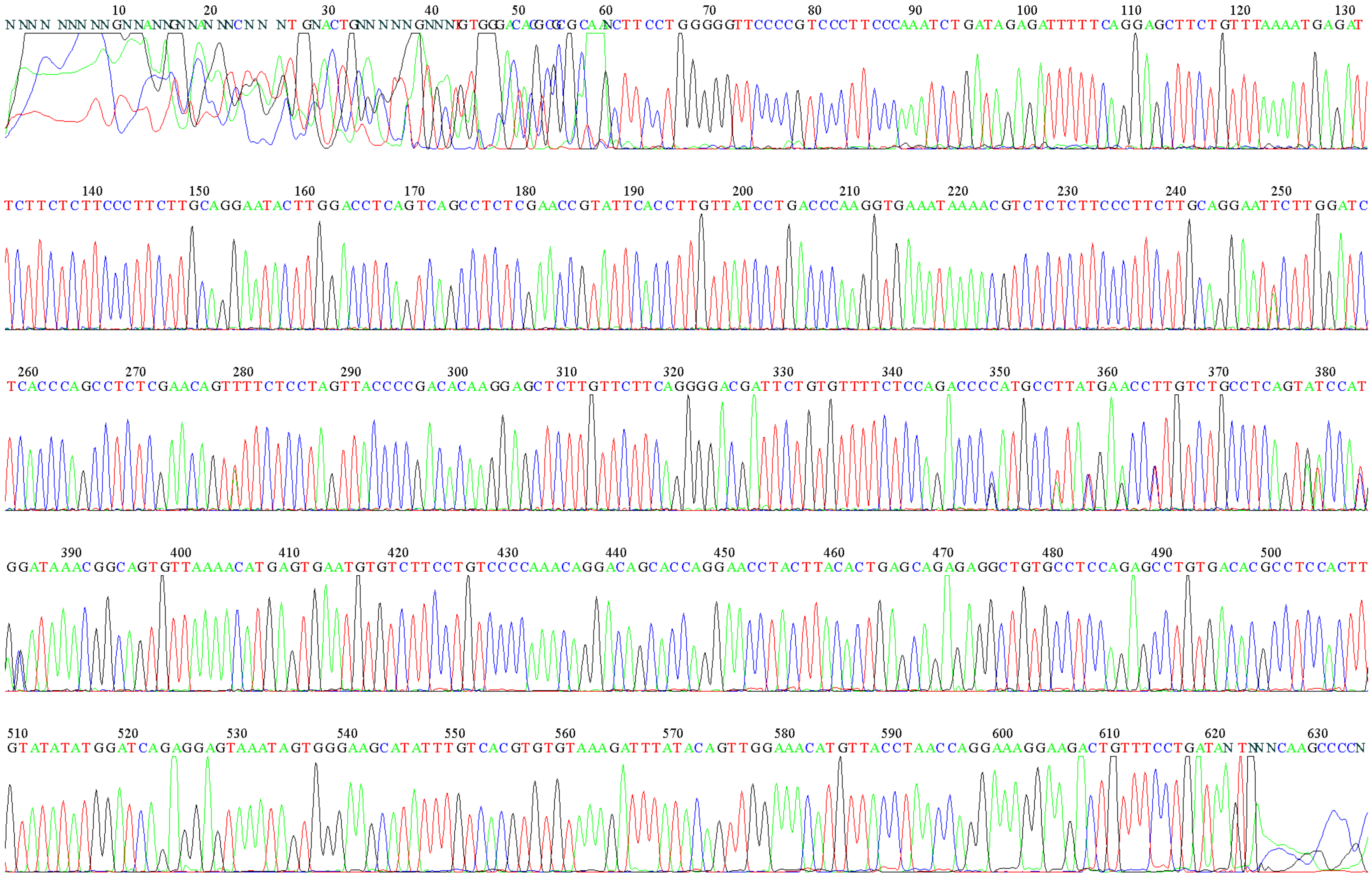

Biology Open • Supplementary information

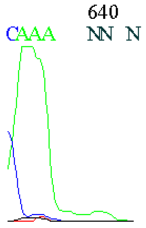


Biology Open • Supplementary information

**Dataset 2. *Fgfr2$^{FCPGT}$* sequence.**

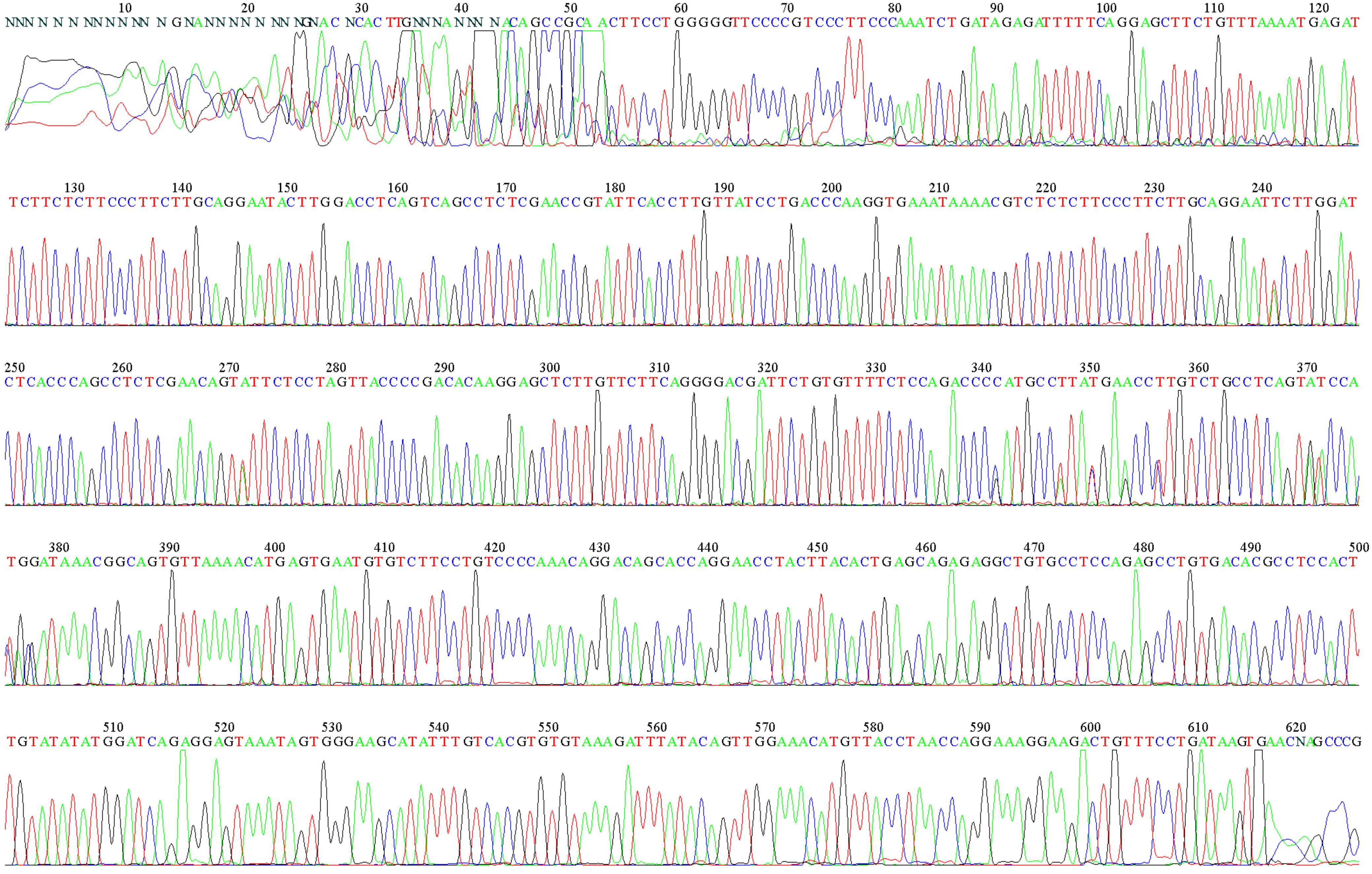

Biology Open • Supplementary information

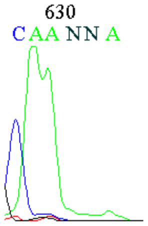


Biology Open • Supplementary information
